# A Case of Puerperal Convulsions—Death

**Published:** 1880-11-20

**Authors:** 


### A CASE OF PUERPERAL CONVULSIONS—DEATH.

We read in a Report by Dr. Isham, of Walnut Hills, in Obstetric Gazette: About 9:15 o'clock, a.m., April 18th, 1880, was called to see Mrs. K., æt. 26, at the end of the seventh month of pregnancy with first child. She had been previously in the best of health, and had suffered none of the usual discomforts incident to her condition. She complained of violent peculiar headache about the frontal region, of extreme nervousness, and she was retching every few minutes, occasionally bringing up a mouthful or two of watery fluid, having a greenish, yellowish tinge. There was great restlessness; the patient demanded constant relief; her manner was impatient and curt. This was in marked contrast to her usual deportment, which was ever gentle and considerate, even upon occasions when suffering quite severely from attacks of supra orbital neuralgia.

One-fifth grain of morphia sulph. and thirty minims of spirits of camphor were administered to ease the pain and allay the stomach irritability. After this she became tranquil, expressed herself as feeling greatly relieved, and she dozed very placidly between intervals of retching, which happened once in about every fifteen minutes. At 10:30 a.m. she awoke from a quiet sleep, raised her head as though desiring the slop-bowl, and went into the most terrific convulsion. The body and limbs became rigid; the limbs were jerked irregularly; the head was drawn to the left side; there were spasmodic twitchings of the eyelids and facial muscles; spasmodic rotatory and oblique movements of the eyeballs; the tongue was bitten and frothy; bloody saliva was ejected from the mouth. This was followed by unconsciousness and stertorous blowing respiration. Having no instruments at hand for phlebotomy, Dr. Geyt was sent for with a request to bring appropriate instruments. He responded promptly, and, with his aid and full concurrence, one quart of blood was drawn—all that would flow from the incised vessel. This considerably abated the severity of the spasms, although they occurred every half hour, and the patient became semi-conscious. By an affirmative nod she signified a willingness to take medicine, which, as bromide of potassium, was given per orem in doses of ʒ ss. at short intervals. Very soon, however, there was a relapse to complete coma, and the convulsions gradually increased in intensity. At the suggestion of the friends, Dr. Reamy was called in consultation, arriving about 3 o'clock p.m. The rectum was washed out with salt and water, and ʒ ss. chloral hydrate, which had been held in abeyance, awaiting his arrival, was then administered per rectum, and chlorororm inhalations were practiced to ward off the convulsions. The chloral lengthened the interval between the seizures. It was questionable if the chloroform was of any advantage, since the patient passed at once from the quiet of coma into a convulsed state without any premonition, except a rolling up of the eyeballs under slightly opened lids. At most, it only shortened the duration of the attacks, perhaps, once or twice, prevented them. The urine drawn with the catheter was highly albuminous. At 8 o'clock p.m. another chloral injection was given, and again at 11 o'clock p.m. The lengthening of the interval between convulsions was marked after the exhibition of this agent.

At no time was there any indication of uterine action. During the convulsions the os uteri would dilate to the size of a quarter of a dollar, but it closed up again after the convulsive action had passed off. There being no improvement in the condition of the patient, it was concluded to induce labor as the only recourse promising any amelioration in the case. Accordingly, about midnight, Dr. Reamy ruptured the membranes, and proceeded to dilate the os with his hand, while I kept the subject fully under the influence of chloroform. There was an indisposition to yield, and only after long efforts at dilatation on the part of both of us, was a diameter of three inches gained. Beyond this the structure would not relax. Dr. Reamy then applied the forceps, and made careful endeavors to bring the head through, but without avail. The convulsive action having cut off the fœtal circulation, he then performed craniotomy, and effected delivery of a five-pound fœtus through the small aperture by means of the forceps. Delivery was completed about 3:30 a.m., the morning of the 19th, and from beginning to termination was artificial, without a uterine contraction worthy of mention. All medication was suspended from this time. About 5 o'clock a.m., it was evident that rupture of the middle cerebral artery had occurred from cessation of general convulsive movement, with peralysis of the right side of the body, and spasmodic action of the muscles of the left leg and arm. There were also movements of both eyeballs laterally and upwards beneath the closed lids.

At 7 a.m., ecchymosed spots were apparent upon the face and neck; the skin assumed a livid hue; the pulse became thready; paralysis was complete upon both sides, and there was involuntary passage of feces and urine. The breathing was the see-saw of shallow respiration, with occasional interrupted gasps like the final acts. Nevertheless, the play of life kept up—sometimes with pretty distinct pulse and fair respiration, and again pulseless with irregular superficial respiration, until about 4 p.m. of the 20th, when it ceased forever.

Observing events in their order, we have first acute cerebral congestion, overwhelming the centers, the seats of consciousness by the pressure upon the cortex irritating the brain mass, and producing the convulsive muscular action. The pressure was somewhat decreased by the venesection reducing the blood volume, as shown by partial return to consciousness. Then it again increased, going on to rupture the middle cerebral artery upon the left side. Evidencing this was the paralysis upon the right side and the localization of spasmodic action to the eyeballs and the extremities of the left side, together with the final complete paralysis with extension of the extravasation. The effusion of blood from the ruptured vessel into the nervous substance of the left cerebral hemisphere paralyzed the centres upon that side governing the movements of the opposite side of the body. It also, by pressure, occasioned irritability of the centres of the right hemisphere, including the tuberculæ quadrigemina, inducing the muscular spasms witnessed upon the left side and the movements of the eyeballs. The cortical pressure was obviated by the local escape of blood, and the presure manifestation limited to the parts innervated from the area of blood extravasation.

Following up the succession of pathological incidents in this case the question of cerebral localization is generally arrived at. While the increased pressure was distributed equally throughout the cerebral vessels, the cortical substance chiefly was irritated, and this irritation was reflected internally to the motor centres, producing the general convulsions. But when the cortical pressure was relieved by the local rupture of the arterial vessel, it is not presumable that the cortex could be subject to future irritation, and the local spasms succeeding the general must have been due to direct irritation of the motor centres themselves—the corpora striata of the thalami and tubercula quadrigemina. The observations in this case, as far as they extend, are in accord with the views of Schiff, that the cortical irritable zones are not motor but sensory centres, through which the sensory fibres pass on their way to the reflex motor centres in the interior.

*Discussion.*—Dr. Henderson said that as regards the treatment of these cases he had an abiding faith in the hypodermic injections of morphia. In puerperal convulsions where assimilation is already impaired, we cannot expect medicine administered per orum to be readily absorbed—rather the contrary; but administered hypodermically, its action is almost immediate. Morphia allays irritation, equalizes the circulation. In the experience of the speaker, neither hydrate of chloral nor potass. bromid. had given satisfaction. The speaker believed that the morphia, to have its beneficial effect, must be given in large doses (hypoderm.), one-half grain at once to quiet the convulsion. Even in cases like the one just reported, where the face becomes livid after the first convulsion, indicating cerebral congestion, he would administer the morphia. It equalizes the circulation and is anti-inflammatory.

Dr. Isham reported that morphia had been the first remedy administered; the patient was fully under its influence. Afterwards symptoms manifested indicating the necessity for a rapid unloading of the vessels.

Dr. C. O. Wright said his experience as regards hydrate of chloral in puerperal convulsions did not coincide with that of Dr. Henderson. He had used hydrate of chloral with the greatest satisfaction and benefit, the convulsions being rapidly controlled thereby.

